# *Dolosigranulum pigrum* keratitis: a three-case series

**DOI:** 10.1186/1471-2415-13-31

**Published:** 2013-07-10

**Authors:** Magali Sampo, Oaufeh Ghazouani, Dominique Cadiou, Elodie Trichet, Louis Hoffart, Michel Drancourt

**Affiliations:** 1 Ophthalmology department, Assistance Publique – Hôpitaux de Marseille, Marseille, France; 2 Bacteriology department, Pôle des Maladies Infectieuses, Assistance Publique – Hôpitaux de Marseille and Unité de Recherche sur les Maladies Infectieuses et Tropicales Emergentes (URMITE), UMR CNRS 7278, IRD 198, INSERM 1095, Faculté de médecine, Marseille, France; 3 Ophthalmology department, Hôpital Saint-Joseph, Marseille, France

**Keywords:** *Dolosigranulum pigrum*, Keratitis, Keratitis kit

## Abstract

**Background:**

*Dolosigranulum pigrum* is a commensal inhabitant of the upper respiratory tract suspected to be responsible for ocular infections but no well-described case of *D. pigrum* corneal infection has been reported. Herein culture and PCR-sequencing-based investigations of corneal scraping specimens confirmed *D. pigrum* keratitis in three patients.

**Case presentation:**

Three elderly patients presented with unilateral keratitis. None was a corneal-contact lens wearer, one had previous cataract surgery and another suffered rheumatoid arthritis sicca syndrome. Culturing the corneal scraping specimen was positive for two cases and PCR-sequencing of bacterial 16S rDNA in the presence of negative controls identified *D. pigrum* in three cases. The two *D. pigrum* isolates were in-vitro susceptible to penicillin G, amoxicillin, doxycycline, rifampicin and gentamicin. In all cases, surgical treatment of corneal thinning was necessary, but corneal perforation occurred in two cases despite intensive antimicrobial treatment with ticarcillin, gentamicin and vancomycin or levofloxacin eye drops leading to enucleation in one case.

**Conclusions:**

*D. pigrum* is the likely cause of corneal infection in three patients, with effective antibiotic treatment in two patients.

## Background

*Dolosigranulum pigrum*, an unusual facultatively anaerobic Gram-positive organism [1] which is part of the oral flora [2], has been rarely implicated as an agent of pneumonia [3,4] including lung infection in cystic fibrosis patients [5], septicaemia [4], cholecystitis [6] and synovitis [7]. While *D. pigrum* has been isolated from the ocular surface [8], no well-described case of *D. pigrum* corneal infection has been described. We herein report three cases of *D. pigrum* keratitis.

## Case presentation

### Case 1

**Figure 1:**
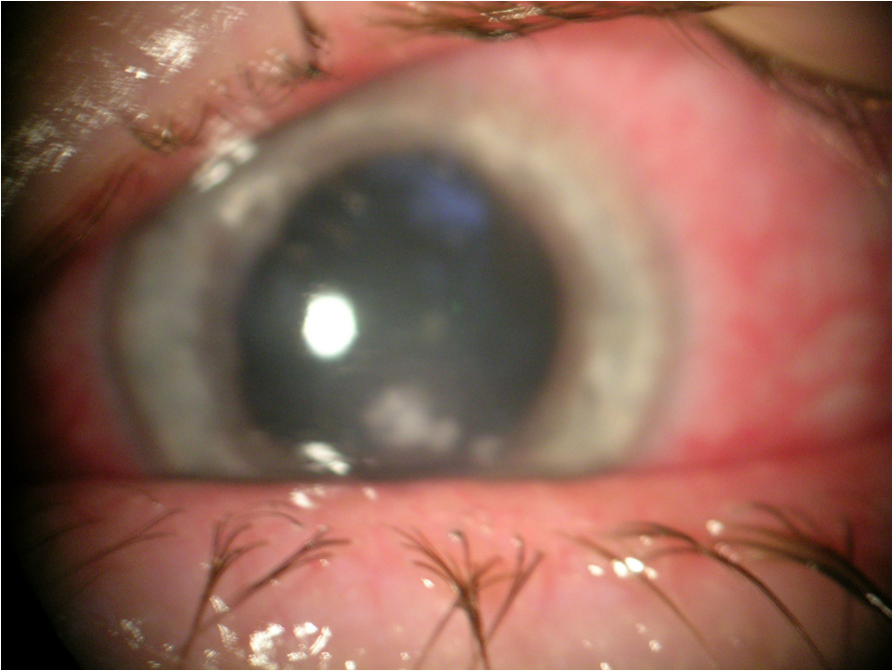
***Dolosigranulum pigrum *keratitis in patient n1.** Central corneal whitish infiltrate at patient’s initial presentation.

A 78-year-old female patient presented to our ophthalmology department in September 2007 for a painful right eye. There was no history of associated diseases in medical records. Slit lamp examination found conjunctival hyperaemia associated with a central corneal whitish infiltrate (Figure 1). The patient had been given topical norfloxacin 3 mg/mL QID prior to admission in our department. As herpetic keratitis was suspected, the patient was treated with 3 g/day oral valacyclovir but the corneal ulcer progressively increased in depth. Despite further intensive treatment by topical antibiotics (levofloxacin 5 mg/mL, gentamicin 14 mg/mL and vancomycin 50 mg/mL eye drops hourly), a corneal paracentral perforation occurred. Fungal keratitis was then clinically suspected and the patient was treated with local amphotericine B 2 mg/mL and oral itraconazole, 200 mg/day. Surgical treatment by Gore-tex patch suturing and a further amniotic membrane graft was done to close the corneal perforation. The patient was then treated by 500 mg/day oral levofloxacin and topical dexamethasone 1 mg/mL without any success. Then a choroidal detachment related to ocular hypotonia led to total visual loss. An evisceration was performed 3 months later related to chronic ocular pain.

At first examination, a corneal swab and a corneal scraping was made as part as a “keratitis kit” (Figure 1) and remained sterile after 10-day incubation at 37°C on 5% sheep-blood agar. In the meantime, additional microbiological analyses including fungal culture and the molecular detection of amoeba, mycobacteria and herpes simplex virus (based on the 18S rDNA, *rpo*B and DNA polymerase genes, respectively) were negative. Ten days after initial examination, the corneal scraping however tested positive for 16S rDNA in the presence of negative controls and sequencing the 16S rDNA PCR product yielded 982/990 bases (99%) and 881/881 bases (100%) sequence similarity with the homologous sequence of *D. pigrum* reference strain (GenBank X70907) and with *D. pigrum* clone 6113702 (GenBank EF517957.1) and only 93% with the following identified species *Alloiococcus otitis*.

### Case 2

A 85-year-man underwent cataract extraction of his right eye in December 2011 by phacoemulsification with posterior chamber IOl implantation through a superior clear corneal incision. 1 mg cefuroxime intracameral injection was performed at the end of the surgery. Postoperative treatment included topical tobramycine 3 mg/mL and dexamethasone 1 mg/mL TID for 4 weeks. The one-month follow-up found a central corneal abscess that led to hospitalization. Corneal scrapings were obtained after topical anaesthesia as part as a “keratitis kit” and an intensive treatment by ticarcillin 6 mg/mL, gentamicin 30 mg/mL and vancomycin 50 mg/mL eye drops hourly was introduced. The corneal ulcer increased in depth and an amniotic membrane graft was performed. The patient was then treated with topical ticarcillin 6 mg/mL combined with topical vancomycin 50 mg/mL QID for 2 days. The outcome was favourable and the patient left the service with ciprofloxacine 3 mg/mL eye drops QID. One-month follow-up showed no recurrence of infection and a 20/125 best corrected visual acuity on right eye. The corneal scraping yielded a gram-positive bacillus after 4-day incubation at 37°C on 5% sheep-blood agar under anaerobic atmosphere. Sequencing the 16S rDNA PCR product obtained from the isolate yielded 988/990 bases (99.8%) sequence similarity with the homologous sequence of *D. pigrum* reference strain (GenBank X70907). The minimum inhibitory concentration (MIC) determined by the disk diffusion method was < 0.25 mg/L for penicillin G, < 0.06 mg/L for amoxicillin, < 0.5 mg/L for doxycycline, < 0.25 for rifampicin and < 128 mg/L for gentamicin 500; the isolate was resistant to eryhromycin (MIC > 1 mg/L). In the meantime, additional microbiological analyses including fungal culture and the molecular detection of amoeba, bacteria, mycobacteria and herpes simplex virus (based on the 18S rDNA, 16S rDNA, *rpo*B and DNA polymerase genes, respectively) were negative.

### Case 3

A 71-year-female with history of rheumatoid arthritis came for a painful right eye with decreased visual acuity for one week. Rheumatoid-associated ocular dryness was treated by preservative-free hyaluronic acid eye drops. Despite topical non-steroidal anti-inflammatory drop (Indometacine, Chauvin, Montpellier, France), no improvement was seen and the patient came in our ophthalmology department one week later in February 2012. Initial visual acuity was limited to “hand movement”. The initial slit-lamp examination found a central corneal whitish infiltrate associated wit severe corneal thinning and hypopyon. The corneal scraping was performed as part as a “keratitis kit” on her arrival in the service. Examination remains stable after ticarcillin 6 mg/mL, gentamicin 14 mg/mL and vancomycin 50 mg/mL eye drops hourly for one day. A central keratolysis associated with rheumatoid arthritis was suspected and a local treatment by topical dexamethasone 1 mg/mL was introduced. Initially, evolution was favourable with disappearance of hypopyon, but four days later, perforation occurred, treated with fibrin-glue. After corneal epithelial healing, the patient was treated with topical dexamethasone 1 mg/mL and neomycin 5 mg/mL TID. Biomicroscopy at one month showed a healing ulcer and a visual acuity limited to light perception was observed.

The corneal scraping yielded a gram-positive bacillus after 3-day incubation at 37°C on 5% sheep-blood agar under anaerobic atmosphere. Sequencing the 16S rDNA PCR product obtained from the isolate yielded 986/990 bases (99.7%) sequence similarity with the homologous sequence of *D. pigrum* reference strain (GenBank X70907). The MIC determined by the disk-diffusion method was of < 0.125 mg/L for penicillin G, < 0.25 mg/L for amoxicillin, < 0.25 mg/L for doxycycline, < 0.5 for rifampicin and < 64 mg/L for gentamicin 500; the isolate was resistant to eryhromycin (MIC > 1 mg/L). In the meantime, additional microbiological analyses including fungal culture and the molecular detection of amoeba, bacteria, mycobacteria and herpes simplex virus (based on the 18S rDNA, 16S rDNA, *rpo*B and DNA polymerase genes, respectively) were negative.

## Conclusions

In these cases, *D. pigrum* was firmly identified by PCR-sequencing of the bacterial 16S rDNA since negative controls remained negative and no other *D. pigrum* strain was isolated or PCR-amplified in the laboratory during the previous weeks while it is now well established that the universal 16S rRNA gene-based detection of bacteria may lack sensitivity compared to culture for antibiotic-free specimens, as illustrated in two cases [9]. The failure to isolate the organism from the same specimen where *D. pigrum* DNA was PCR-amplified in one patient may be due to the inhibitory effect on growth of local antibiotic treatment prior to corneal sampling. Indeed, *D. pigrum* has been shown to be *in-vitro* susceptible to antibiotics used in these patients, including beta-lactams, fluoroquinolones and vancomycin [8].

Despite the fact that no organism was observed at direct microscopic observation of the clinical specimens, the *D. pigrum* organism herein detected was most likely a causal agent of keratitis in these patients because of no other known keratitis agent has been detected despite extensive culture-based and PCR-based laboratory investigations. They were three elderly patients who did not wear corneal contact lens. Potential risk factors included a recent cataract extraction in one patient (case n° 2) and rheumatoïd arthritis-sicca syndrome in another patient (case n° 3). These observations suggest that *D. pigrum* keratitis may follow minor corneal trauma. Apart from a long, potentially unfavourable course, we found no sign or symptom that was particularly associated with *D. pigrum* keratitis.

A previous report recovered *D. pigrum* from six eye infections, including one case of blepharitis. All were erythromycin-resistant. The authors conclude that *D. pigrum* has the capability to cause eye infections [8]. Furthermore, one of two isolates which supported the initial description of *D. pigrum*, was isolated in 1991 from a bandage corneal contact lens and an eye swab from a patient with neurotrophic cornea complaining from discomfort and blurred vision [2]. However, no well-described case report of *D. pigrum* keratitis has been previously published and we herein report on the first case series. The three patients here reported have been observed among 500 keratitis patients over 5 years.

**Figure 2:**
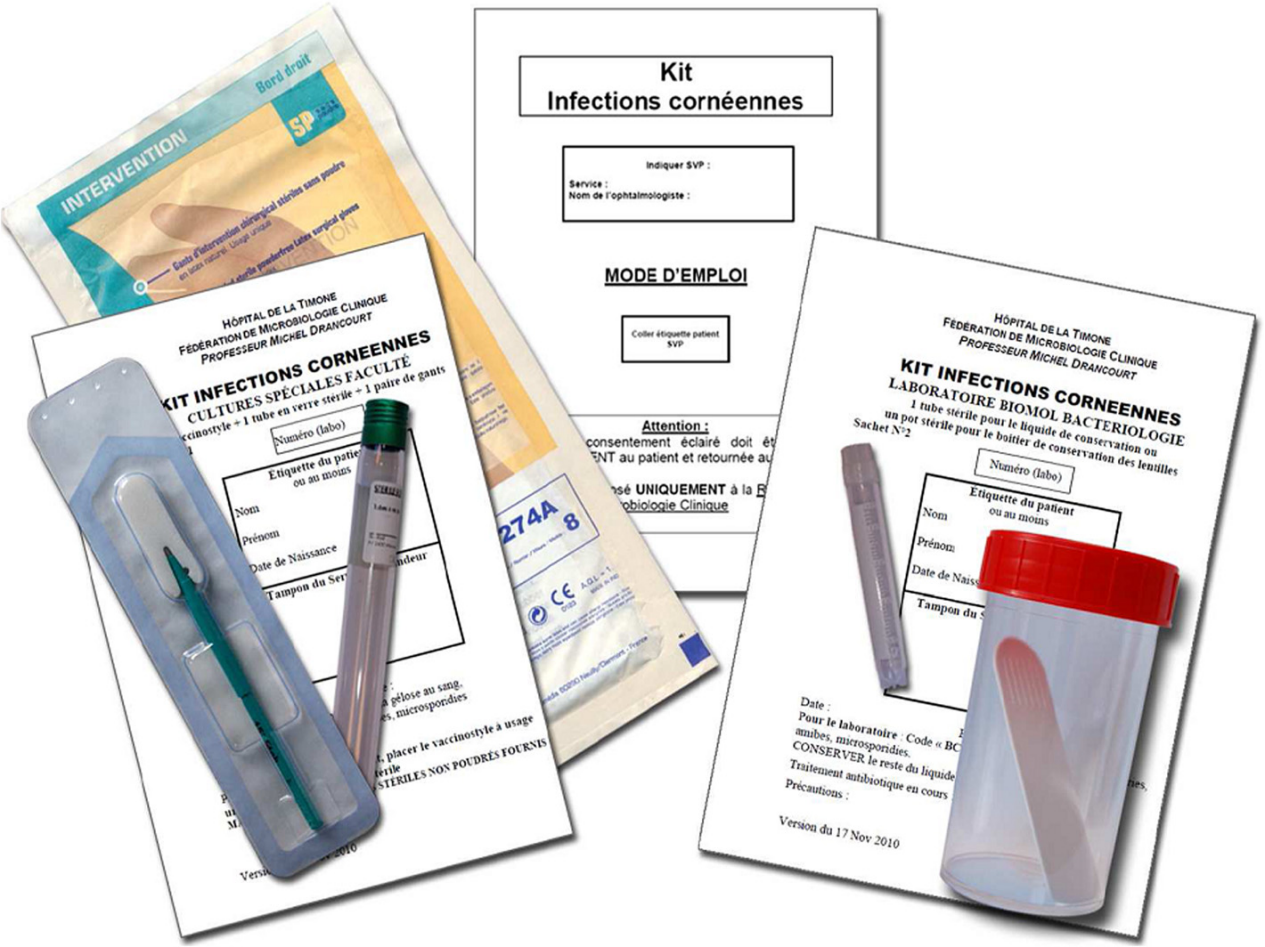
**The “keratitis kit” aims to standardize specimen collection and their laboratory manipulation.** It comprises of pre-labelled laboratory forms and patient informed consent, a vaccinostyle for corneal scraping and a tube for serum collection. In the laboratory, vaccinostyle is inoculated on 5% blood agar both anaerobically and under 5% CO_2_ atmosphere, and on amoeba. The serum is tested for common bacterial keratitis.

These cases illustrate that *D. pigrum* may be an emerging pathogen. Our experience also suggests that this unusual species may target elderly patients and produce a progressive and destructive course. A standardised “keratitis kit” (Figure 2) allows for systematic molecular testing of corneal specimen and the detection of anaerobes such as *D. pigrum* as reported in these three patients.

## Consent

Written informed consent was obtained from the patients for publication of these case reports and any accompanying images. A copy of the written consent is available for review by the Editor-in-Chief of this journal.

## Competing interests

The authors declare that they have no competing interests.

## Authors’ contributions

MS, ET, DC and LH provided the clinical care of the reported patients and all were involved to draft the manuscript. OG and MD were involved in establishing the microbiological diagnosis. MD and LH conceived of the study, and participated in its design and coordination and helped to draft the manuscript. All authors read and approved the final version of the manuscript.

## Pre-publication history

The pre-publication history for this paper can be accessed here:

http://www.biomedcentral.com/1471-2415/13/31/prepub
